# Fork head transcription factor is required for ovarian mature in the brown planthopper, *Nilaparvata lugens *(Stål)

**DOI:** 10.1186/1471-2199-12-53

**Published:** 2011-12-31

**Authors:** Xiaolin Dong, Yifan Zhai, Jianqing Zhang, Zhongxiang Sun, Jing Chen, Jie Chen, Wenqing Zhang

**Affiliations:** 1 School of Life Sciences, Sun Yat-sen University, Guangzhou, Guangdong, 510275 China

## Abstract

**Background:**

The brown planthopper (BPH), *Nilaparvata lugens*, is the most devastating rice pest in many areas throughout Asia. The reproductive system of female *N. lugens *consists of a pair of ovaries with 24-33 ovarioles per ovary in most individuals which determine its fecundity. The fork head (Fox) is a transcriptional regulatory molecule, which regulates and controls many physiological processes in eukaryotes. The Fox family has several subclasses and members, and several Fox factors have been reported to be involved in regulating fecundity.

**Results:**

We have cloned a fork head gene in *N. lugens*. The full-length cDNA of *Nl*FoxA is 1789 bp and has an open reading frame of 1143 bp, encoding a protein of 380 amino acids. Quantitative real-time PCR (RT-qPCR) and Reverse Transcription- PCR (RT-PCR) analysis revealed that *NlFoxA *mRNA was mainly expressed in the fat body, midgut, cuticle and Malpighian tube, and was expressed continuously with little change during all the developmental stages. *Nl*FoxA belongs to the FoxA subfamily of the Fox transcription factors. Knockdown of *NlFoxA *expression by RNAi using artificial diet containing double-stranded RNA (dsRNA) significantly decreased the number of offspring and impacted the development of ovaries. ELISA and Western blot analyses showed that feeding-based RNAi of *NlFoxA *gene also resulted in decreased expression of vitellogenin (Vg) protein.

**Conclusion:**

*Nl*FoxA plays an important role in regulation of fecundity and development of ovaries in the BPH via regulating vitellogenin expression.

## Background

The brown planthopper (BPH), *Nilaparvata lugens*, is a major pest to rice production in many areas of Asia. It shows two wing forms, long (macropterous) and short (brachypterous) ones, in its adult stage. The macropterous adults possess the ability to migrate across long distances, while the brachypterous adults exemplify strong reproductive capacities. One individual brachypterous female has the ability to produce a large number of offspring, increasing each generation size by 10- to 40-folds [1,2]. The macropterous adults migrate long distances every year to the rice-growing areas of China, Japan and Korea, resulting in severe infestations [3,4], causing hopperburn and ultimately leading to reductions in rice yields. The BPH ingests nutriments specifically from the phloem of rice plants with its stylet, causing whole plants to become yellow and rapidly dry, a phenomenon referred to as hopperburn [5]. In additional, BPH is a vector for some rice diseases such as the rice ragged stunt virus (RRSV) and rice grassy stunt virus (RGSV) [6]. BPH often causes losses of up to 60% in rice crop yields [7]. In China, *N. lugens *covered areas totaling 9.3 million hm^2 ^in 1974, and in 2005, the affected area has increased to 23.23 million hm^2^, causing huge economic losses [8]. Because insecticides have been extensively used to control this pest, resistances have begun to arise in different countries and areas [9-11]. This pattern of resistance causes ecological imbalances in predator-prey relationships and has often resulted in the resurgence of BPH [12]. Despite the extensive literature documenting the effects of host plant quality on the performance of herbivorous insects, surprisingly few publications have considered the fecundity of the BPH.

The reproductive system of female insects consists of a pair of ovaries, ovarioles is the function units of the ovary containing a series of tapering egg tubes. There is a progression of developing oocytes in the ovarioles. The number of ovarioles in each ovary varies tremendously in different insect species and determines fecundity [13]. The *N. lugens *has 24-33 ovarioles per ovary in most individuals [14].

The fork head (Fox) family transcription factors have several subclasses and members, designated as A to Q [15,16], and they share a structurally conservative fork head box defined by a 'winged helix' DNA-binding domain [16]. The first fork head protein, FoxA, was first found in *D. melanogaster *and importantly required in the embryonic development [17]. Subsequently, several additional Fox transcription factors have been described in various organisms [18]. It has been reported that Fox transcription factors play critical roles in regulation of metabolism, proliferation, apoptosis, development, organogenesis, differentiation and control of oxidative stress through both activation and repression of target gene expression by multiple mechanisms [18-21]. In some insects, Fox factors participate in the regulation of many physiological processes [22-24]. Reports have attested that endocrine hormones play an important role in the development of ovaries [25]. Several Fox factors have been shown to be involved in regulating vitellogenesis, fecundity and ovarian events such as follicular development and selection, ovarian cell proliferation and cancerogenesis, ovarian cell apoptosis, ovarian secretory activity and oocyte/cumulus maturation [26,27]. However, whether FoxA influences reproduction or not is still unknown.

RNA interference (RNAi) through double-stranded RNA (dsRNA) has been used widely to study gene function in insects. Since RNAi was first discovered in the nematode *Caenorhabditis elegans *[28]. RNAi has extensively applied in ovarian function study [29]. Many means of transporting dsRNA into the body of an organism have been explored, including microinjection, soaking, oral feeding and transgenic plant expression [30-34]. Recently, it has been reported that gene knockdown in several insects can be achieved successfully through feeding the insects bacteria that express dsRNA [34,35] or artificial diet containing dsRNA [36]. Directly feeding dsRNA is a simple manipulation, inflicts no body injury and has been established for insect research.

The present study was designed to characterize and identify expression of the transcription factor FoxA in Hemiptera, *N. lugens*. We used RNAi technique to knockdown expression of *Nl*FoxA by feeding the insects an artificial diet containing dsRNA. We also intended to provide evidences to confirm whether FoxA influences reproduction of *N. lugens *and whether it is involved in fundamental biological phenomena and agricultural problems related to the BPH.

## Results

### Isolation and characterization of *NlFoxA *cDNA

**Figure 1:**
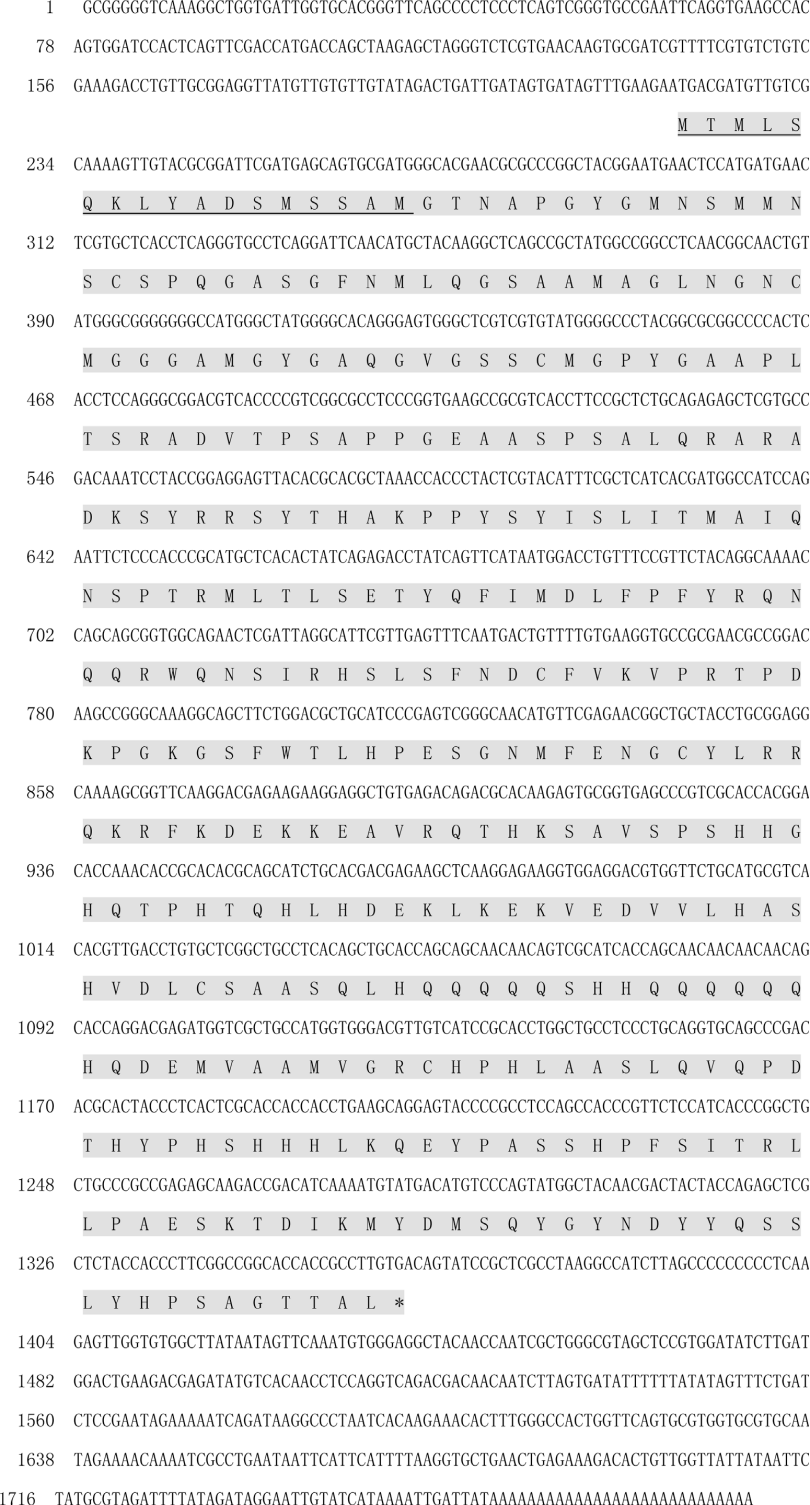
**Nucleotide and deduced amino acid sequences of FoxA from *N. lugens***. "*" terminal codon; underline: predicted signal peptide.

Based on the conserved sequence of fork head from *T. castaneum*, *D. melanogaster *and *B. mori*, a 434-bp cDNA fragment was obtained by homologous cloning using cDNA from the 2^nd ^day of brachypterous female adults as a template. Sequence analysis showed that the deduced amino acid sequence of the cDNA fragment had 41-45% identity to the corresponding region of FoxA from *T. castaneum*, *D. melanogaster *and *B. mori*. Then, the 5'- and 3'-RACE were performed with two pairs of specific primers designed based on the nucleotide sequence of the cDNA fragment. A full-length *NlFoxA *of 1789 bp (GenBank accession No: JF345255) was obtained by overlapping the RACE fragments and the cDNA fragment. Sequence analysis showed that the open reading frame (ORF) of *Nl*FoxA is 1143 bp, encoding 380 amino acid residues with a predicted MW of 42.2 kDa and a pI of 8.6, and there is a 5'-untranslated region (UTR) of 244 bp and a 3'-UTR of 453 bp (Figure 1). An 18-residue signal peptide at the N-terminus was identified by SignalP, thus the mature protein (362 amino acids) has a calculated molecular mass of 40.3 kDa and an estimated pI of 8.6. BLAST searches using *Nl*FoxA as a query showed that all the predicted FoxA proteins, including *Nl*FoxA, from insects and vertebrates contain only one Fox DNA-binding domain, indicating that *Nl*FoxA belonged to the Fox DNA-binding superfamily. *Nl*FoxA contained typical amino acids within the 110-aa-long fkh domain, suggesting that it encodes FoxA [37].

### Developmental and tissue specific expression of *NlFoxA*

**Figure 2:**
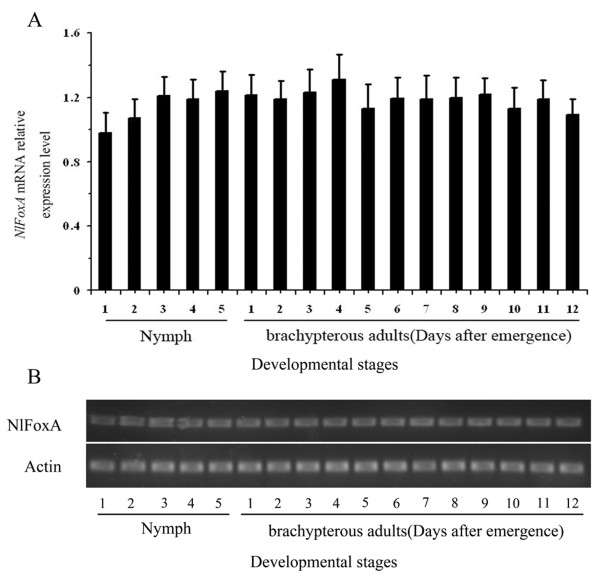
**Expression of *NlFoxA *gene at different developmental stages was determined by RT-qPCR (A) and RT-PCR (B)**. The mRNA level was normalized relative to the β-ACTIN transcript. Each point represents mean value ± S.E.M of three independent experiments with three individuals in each replicate.

**Figure 3:**
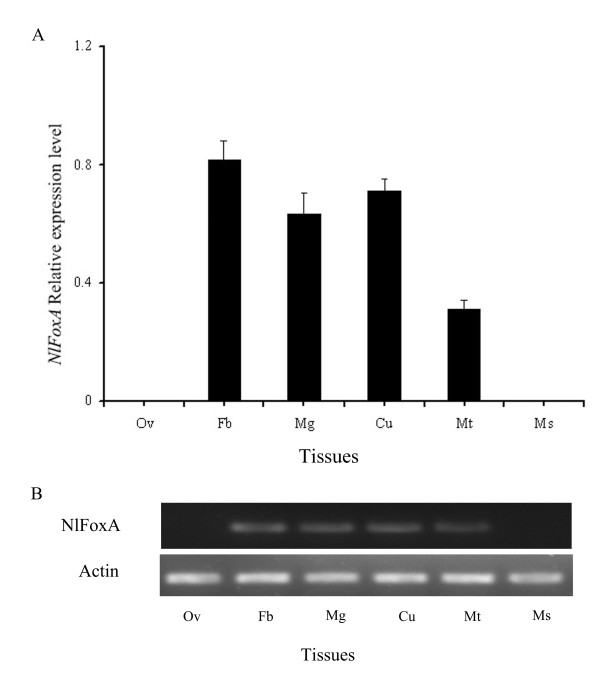
**Expression of *NlFoxA *gene in various tissues of brachypterous adults was determined by RT-qPCR(A) and RT-PCR(B)**. ov, ovaries; fb, fat body; mg, midgut; ms, malpighian tube; cu, cuticles; mc, thoracic muscle. The mRNA level was normalized relative to the β-ACTIN transcript. Each point represents mean value ± S.E.M of three independent experiments with three individuals in each replicate.

To determine whether *NlFoxA *is present during developmental stages and in various tissues in the 3^rd ^day of brachypterous female adult *N. lugens*, total RNA of each sample was isolated. We used RT-qPCR and RT-PCR to characterize the pattern of developmental expression of *NlFoxA *gene for all developmental stages (including nymphs from the 1^st ^to 5^th ^instars and 1-12 days brachypterous female adults). The results demonstrated that *NlFoxA *mRNA was expressed at constantly low levels with little change during developmental stages (Figure 2A &2B). A high level of *NlFoxA *expression was detected in the fat body, midgut, cuticle and the Malpighian tube, but was not found in the ovary and thoracic muscle (Figure 3A &3B).

### Efficiency analyses after ingestion of ds*NlFoxA*

After feeding synchronous nymph groups on 0.5 μg/μl *NlFoxA *dsRNA, dsGFP and the blank control from the 3^rd ^to the 5^th ^instar (10 days), the survival rates were 78.52%, 82.22% and 82.96%, respectively. To investigate the efficiency of RNAi after ingestion of ds *NlFoxA *in *N. lugens*, *NlFoxA *mRNA relative levels were measured by RT-qPCR in brachypterous adults collected 1-8 days after emergence on rice plants.

**Figure 4:**
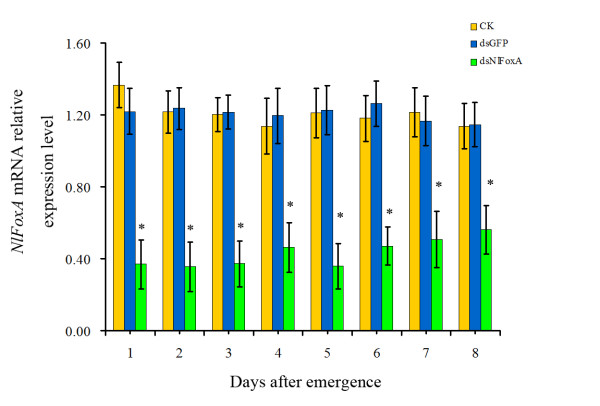
**Detection of the efficiency of feeding-based RNAi and impact on *NlFoxA *mRNA level by RT-qPCR**. The expression levels of *N. lugens FoxA *mRNA after different treatments. CK, control; dsGFP, 0.5 μg/μlds *GFP*; ds *Nl*FoxA, 0.5 μg/μl ds *NlFoxA*, respectively. The data represent the mean values ± S.E.M of three replicates. '*'means statistically significant difference in expression levels between CK and ds *Nl*FoxA(*t*-test, p < 0.05).

The transcript levels of *NlFoxA *were decreased by 55.34%-61.28% from the emergence of brachypterous adults up to the 8^th ^day of adults compared to the blank control (Figure 4). This result confirmed that RNAi-mediated knockdown of *NlFoxA *was highly effective.

### Knockdown of *NlFoxA *or *NlVg *reduces fecundity and disrupts ovarian development

**Figure 5:**
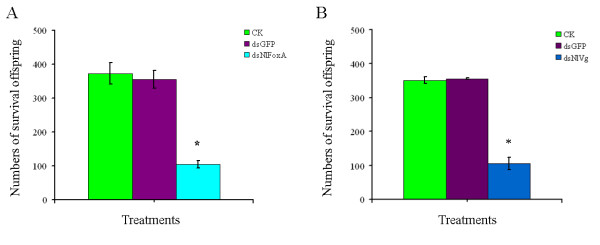
**RNAi-mediated knockdown of *NlFoxA *(A) or *NlVg*(B) gene decrease offspring (eighteen pairs were analyzed per group)**. CK, control; dsGFP, 0.5 μg/μlds *GFP*; ds *Nl*FoxA, 0.5 μg/μl ds *NlFoxA*; ds *Nl*Vg, 0.5 μg/μl ds *NlVg*, respectively. The data represent the mean values ± S.E.M of three replicates. '*' means statistically significant difference in number of offspring between CK and ds *NlFoxA *(*t*-test, p < 0.05).

Once insects emerged, we successfully allocated them into 18 pairs per group. The number of offspring from every individual brachypterous female adult was counted, and the result demonstrated that *Nl*FoxA plays an important role in the reproduction of *N. lugens*. The average number of offspring in the group treated with 0.5 μg/μl *NlFoxA *dsRNA was 104.68, significantly less than those in the control and dsGFP-treated groups (Figure 5A). Interestingly, knockdown of *Nl*Vg showed similar results to those of the *Nl*FoxA knockdown (Figure 5B).

**Figure 6:**
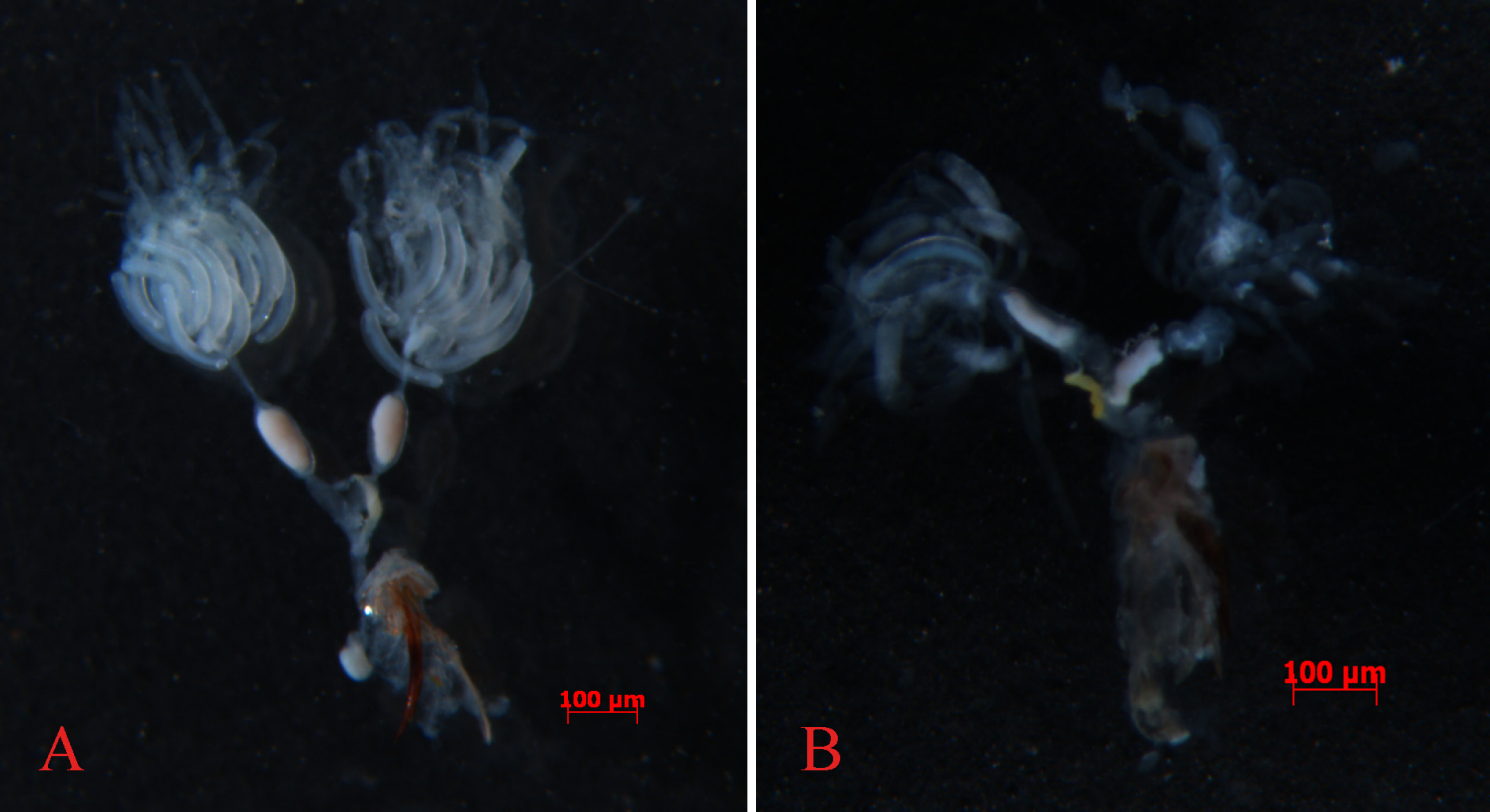
**Ovaries dissected from the 3^rd ^day of brachypterous female adult *N. lugens***. (A) Ovarioles from the control or 0.5 μg/μl dsGFP-treated insects had completely developed ripe eggs. (B) Ovaries from RNAi-treated (0.5 μg/μl ds *NlFoxA *or 0.5 μg/μl ds *Nl Vg *treated) insects with decreased number of ovarioles and fewer eggs.

Although feeding ds*NlFoxA *reduced fecundity, the hatchability rate was unchanged (data not shown). Thus, we set out to determine whether ingestion of ds*NlFoxA *affects the development of ovaries by analysis of morphology. Assessment of ovaries from the 3^rd ^day of brachypterous adults suggested that RNAi knockdown of *NlFoxA *resulted in underdeveloped ovaries with less ovarioles and fewer eggs. In contrast, the control and dsGFP-treated groups showed repletion and no disruption of ovarioles and eggs on the same day (Figure 6). Similar results were obtained when *Nl*Vg was knocked down (data not shown).

### Knockdown of *NlFoxA *results in lower vitellogenin (Vg) gene expression and less Vg protein

**Figure 7:**
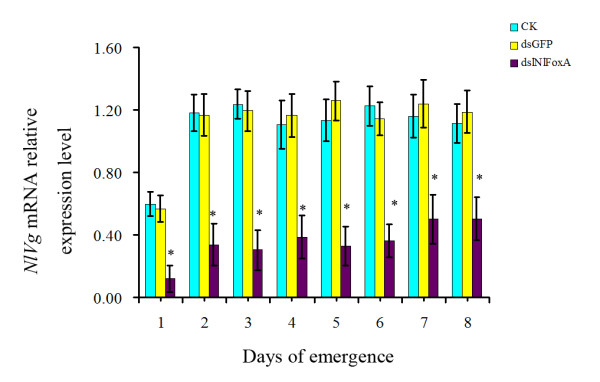
**The relative mRNA expression levels of *Nl*Vg after RNAi of *NlFoxA *gene**. CK, Control; ds *GFP*, 0.5 μg/μlds *GFP*; ds *Nl*FoxA, 0.5 μg/μl *dsNlFoxA *treated. respectively. The data represent the mean values ± S.E.M of three replicates. '*' means statistically significant difference in expression levels between CK and ds *Nl*FoxA (*t*-test, p < 0.05).

Vitellogenin is a conserved yolk precursor protein that is synthesized in the fat body in invertebrates, and the yolk provides nutrition to the developing oocytes for utilization during embryogenesis. We showed that expression of vitellogenin (Vg) gene (GenBank accession No: JF345256) was significantly suppressed after ingestion of ds *NlFoxA *(Figure 7).

**Figure 8:**
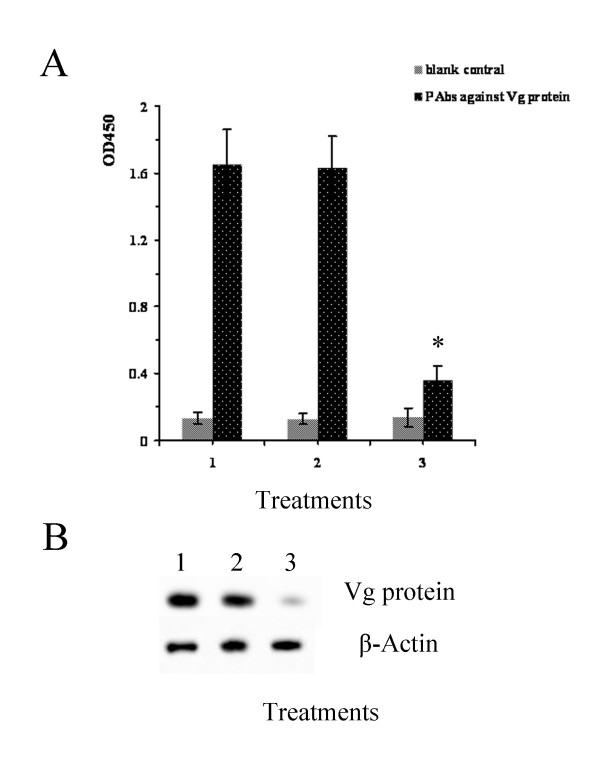
**Differential expression level of Vg proteins in ovariesof BPH**. (A) ELISA analysis. PBST (0.15 M, pH7.4) was used as the blank control. (1) CK, Control, (2) GFP, 0.5 μg/μl ds *GFP*, (3) ds *Nl*FoxA, 0.5 μg/μl ds *NlFoxA*. Data are means ± S.E.M of three independent experiments with fifteen individuals each. '*' means statistically significant difference in OD_450 _values between CK and ds *NlFoxA *(*t*-test, p < 0.05). (B) Western Blot analysis. Immunoblotted with anti-Vg serum (diluted 1:5000) and visualized by ECL. Actin was used as an internal control.

To detect Vg protein in the ovaries of BPH after different treatments, equal amounts of total proteins from three groups were coated to microtiter plates and assayed by indirect ELISA (Figure 8A) and western blotting (Figure 8B) with corresponding primary antibodies. The Vg protein concentration in ovaries of the 3^rd ^day of brachypterous female adults treated with 0.5 μg/μl *NlFoxA *dsRNA was significantly lower than that of the control and dsGFP-treated groups. The increased concentration of *NlFoxA *dsRNA was accompanied with a decreased concentration of Vg protein (data not shown). The putative Vg bands were considerably more intense in the control and dsGFP-treated groups than in the *NlFoxA *dsRNA-treated group, suggesting that *Nl*FoxA regulates ovarian maturity by controlling accumulation of *Nl*Vg in the ovaries.

## Discussion

The brown planthopper fosters a robust ability to produce offspring. One brachypterous adult individual insect can produce more than 400 offspring [1]. This may be the primary cause of damage caused by PBH on rice plants in many areas of Asia. It is commonly known that the macropterous PBH migrate from Vietnam, which is considered the major source of the north-bound migration to China every year [38]. Many factors can affect the fecundity of insects. Host plant quality is a critical factor for herbivorous insects [39]. Endogenous hormones and peptides are also significant components underlying successful reproduction [25,40]. In this study, we investigated how the transcription factor *Nl*FoxA regulates fecundity and ovarian development in *N. lugens*. We first cloned and characterized a cDNA sequence from the Hemiptera insect *N. lugens *encoding FoxA, which shares a high homology with the *T. castaneum *FoxA. Pattern analysis suggests that the expression level of *FoxA *mRNA in N. lugens was almost constant throughout the life cycle (Figure 2A &2B), this maybe because it regulates different physiological processes in specific development phases. The *NlFoxA *was expressed in the fat body, midgut, cuticle and the Malpighian tubes, but was not found in ovaries and thoracic muscle (Figure 3A &3B). The FoxA originates from endoderm and elongates to ectoderm during evolution [41]. In different insects, FoxA is located in different tissues. In *H. armigera*, FoxA was localized to the nuclei of fat body cells [23], but in *Ae. Aegypti*, FoxA was not expressed in fat body but in the thorax, midgut, and malpighian tubules [27]. In *T. castaneum*, it can be detected in yolk nuclei [37]. Our successful cloning of *NlFoxA *not only adds a member to this family of transcription factors but also provides a potential target for the biological control of insect pests.

*Fox *genes encode a family of transcription factors defined by a "winged helix" DNA-binding domain and have been identified in many metazoans, and they play critical roles in regulating diverse physiological processes. In *B. mori*, stabilization of SGF1/fork head to its target sequence is critical to promote fhx transcription at each intermolt [42]. In *D. melanogaster*, fork head controls the timing and tissue selectivity of steroid-induced developmental cell death [43]. In *H. armigera*, FoxA regulates the expression of diapause hormone [23]. In mammals, *FoxA *genes play crucial roles in multiple stages of mammalian life, including early development, organogenesis and differentiation, metabolism and homeostasis [21,44,45]. Fat body is a major tissue for vitellogenin synthesis during vitellogenesis [46]. Studies in American dog tick indicated that Vg uptake is essential for ovarian development [47]. DNA-binding assays revealed that, in *Ae. aegypti*, genomic DNAs containing the 5' upstream region and the positions of regulatory and coding sequences of the mosquito Vg gene are capable of binding to transcription factors [48]. Several transcription factors have been reported to be involved in *Ae. aegypti *fecundity, but the authors did not assess the effect on the reproduction because it is not a 'fat body' Fox factor, and the elaborate mechanisms of these phenotypes were lacking [27]. In this study, we demonstrated that *NlFoxA *expression level was at a constant low level with little change(Figure 2A &2B). Interestingly, downregulation of *NlFoxA *can suppress ovarian development in *N. lugens *(Figure 6) and quickly decrease the number of offspring (Figure 5A). Confirming this effect, the expression levels of *Nl*Vg mRNA (Figure 7) and protein (Figure 8A &8B) were remarkably decreased. Simultaneously, knockdown of *Nl*Vg had similar efficiency (data not shown). In addition, *NlFoxA *is also expressed in the fat body syntopogenic with Vg (Figure 3A &3B). FoxA can bind to the promoters of diapause hormone and pheromone biosynthesis-activating neuropeptide to regulate their expression [23]. Whether *Nl*FoxA regulates *Nl*Vg by directly binding to regulatory elements, inducing hormone synthesis via the other pathway, or supporting the nutritional element of ovary development requires further deliberation.

RNAi has been widely used to study gene function in insects. There are many approaches for delivering dsRNA into an organism's body. It has been reported that gene knockdown in several insects can be achieved successfully through feeding dsRNA. The oral delivery of an artificial diet containing dsRNA to larvae of the lepidopteran species *S. exigua *not only suppressed transcription levels but also led to lethal phenotypes, and proved that SID-1 exists in *S. exigua *[49]. SID-1 is required for spreading RNA-interfering information between tissues, usually leading to systemic RNAi [50]. In a previous paper, we reported that the oral delivery of an artificial diet containing *NlTPS *dsRNA can induce target gene silencing in *N. lugens *[36]. SID-1 has been found in *N. lugens *[51], and *NlFoxA *gene is also expressed in the fat body. Thus, oral delivery of RNA interference can be achieved to knockdown target genes in N. lugens. Further research will address how much dsRNA is required to avoid degradation in the midgut and can reach the target tissues.

## Conclusion

We have demonstrated the existence and characterization of a *FoxA *gene in *N. lugens*, and have shown that oral ingestion of *NlFoxA *dsRNA solutions by *N. lugens *nymphs resulted in decreased expression of the target gene, less fecundity and underdeveloped ovarian in the brachypterous adults via regulating vitellogenin expression.

## Methods

### Insect rearing

The *N. lugens *strain was kindly provided by Guangdong Academy of Agricultural Sciences in September 2007 and has been reared in continuous greenhouse laboratory culture conditions since then on rice plants (Huang Hua Zhan, bought from Guangdong Academy of Agricultural Sciences, Guangzhou, China) [36].

### Cloning and sequence analysis of *NlFoxA*

Total RNA was isolated from four 2^nd ^day brachypterous female adults of *N. lugens *using the Trizol kit (Invitrogen, USA). Its integrity was detected using Agilent 2100 (USA). First-strand cDNA was synthesized with a first strand synthesis kit using reverse transcriptase X L (AMV) and an oligo dT_18 _primer (TaKaRa, Japan). Two pairs of degenerate primers were designed based on the conserved amino acid sequences of fork head from different insects and animals (Table 1). The first-strand cDNA (1 μl) was used as a template for PCR using a general protocol. The reaction mixture contained 0.1 mM dNTP, 0.5 mM of each degenerate primer and 1.0 U of HiFi-Taq DNA polymerase (TransGen Biotech, Guangzhou, China) in a total volume of 25 μl. The first PCR was carried out with the following conditions: initial preheating for 5 min at 94°C, 35 cycles at 94°C for 30 s, 48°C for 30 s and 72°C for 1 min, and with a final extension at 72°C for 10 min using the primer pair *Nl*FoxAF1 and *Nl*FoxAR1. The second PCR was performed using another degenerate pair, *Nl*FoxAF2 and *Nl*FoxAR2, with the aforementioned program. The amplified fragment was recovered in a 1% agarose gel and purified using the Gel Extraction Kit (Omega, USA). Purified DNA was ligated into the pMD18-T vector (TaKaRa, Japan), and recombinant clones were digested with *EcoR*I and *Pst*I to screen the presence of inserted DNA. Positive clones were sequenced by Invitrogen company (Shanghai, China). To obtain the full-length *NlFoxA *cDNA, we used a RACE Kit (CLONTECH, Japan). Specific primers for the 5'- and 3'- Rapid Amplification of cDNA Ends (RACE) were designed based on homologous PCR fragments. The specific primers 5*Nl*FoxA1 and 5*Nl*FoxA2 were used for 5^'^-RACE, while 3*Nl*FoxA1 and 3*Nl*FoxA2 were used for 3^'^-RACE (Table 1). Using the 5'- and 3'-RACE cDNAs as templates, PCR was performed using the 5*Nl*FoxA1 primer and Universal Primer Mix (UPM, Clontech) by denaturing at 95°C for 30 s, followed by 35 cycles of 95°C for 30 s, 55°C for 30 s and 72°C for 2 min, and a final extension at 72°C for 10 min. Nested PCR was carried out with the first-round PCR product as a template and the Nested Universal Primer A (NUP, Clontech) and *Nl*FoxA2 primer. The reaction conditions consisted of the followings: 6 min of initial preheating at 94°C, 30 cycles of 94°C for 30 s, 68°C for 30 s and 72°C for 40 s, and a final elongation at 72°C for 7 min. The RACE products were purified and sequenced as described above. Sequence homologous alignment and similarity searches were carried out by Blast biological software http://www.ncbi.nlm.nih.gov/blast. The signal peptide was analyzed by the SignalP procedure http://www.expasy.ch/SignalP.

**Table 1:** Primers used in this study

Primers	Primer sequence
Degenerate primers	
*Nl*FoxAF1	5-CACGCSAAGCCBCCVTACTC-3
*Nl*FoxAF2	5-ATCACSATGGCBATMCAGAA-3
*Nl*FoxAR1	5-TCYTTYTTYTCGTCYTTGAA-3
*Nl*FoxAR2	5-CCGTTYTCRAACATGTTVCC-3
For RACE	
3*Nl*FoxA1	5'-AGTTTCAATGACTGTTTTGTGAA-3'
3*Nl*FoxA2	5'-TTCTGGACSCTGCATCCCGAGT-3'
5*Nl*FoxA1	5'-AGAATTCTGGATRGCCATCG-3'
5*Nl*FoxA2	5'-GATARGTCTCTGATAGTGTGA-3'
For RT-PCR and real-time PCR	
Q *Nl*FoxAF	5'-TTACACGCACGCTAAACCAC-3'
Q *Nl*FoxAR	5'-CCTCATCAGCCCAAGGGAACAA-3'
Q *Nl*VgF	5'-GCATCAATGAACCCAGCTAACTC-3'
Q *Nl*VgR	5'-TGGACGGCTCTTTGCATACTCC-3'
ACTIN-F	5'-TGCGTGACATCAAGGAGAAGC-3'
ACTIN-R	5'-CGGCACCTTCACAAAACAG-3'
For *Nl*FoxA dsRNA synthesis	
*Nl*FoxA -F	5'-TTCGAGAACGGCTGCTACCTGCGG-3'
*Nl*FoxA -R	5'-GCTCTCGGCGGGCAGCAGCC-3'
For GFP dsRNA synthesis	
GFP-F	5'-AAGGGCGAGGAGCTGTTCACCG-3'
GFP-R	5'-CAGCAGGACCATGTGATCGCGC-3'
For *Nl*Vg protein expression	
*Nl*Vg-EF	5'-GTA **CCATGG **CCAGTAACT TCCCCAATG TG-3'
*Nl*Vg-ER	5'-GAT **CTCGAG**CTTGGCCAAGACAACAACCTTC-3'
For *Nl*Vg dsRNA synthesis	
*Nl*Vg -F	5'-ACAGCCAGTCCAACAGCTTCTAC-3'
*Nl*Vg-R	5'-TGCTGCTGCTGCTGCTGCTTC-3'

### Quantitative real-time PCR (RT-qPCR)and Reverse transcriptase PCR (RT-PCR)

The expression pattern of *FoxA *in *N. lugens *was detected by RT-qPCR using a Light Cycler 480 system (Roche Diagnostics Indianapolis, IN, USA) and SYBR Premix *Ex *Taq (Takara, Japan). Briefly, the copy number of the target genes and C_T _values were negative correlated. It means that one sample containing a higher number of copies of the target gene had a lower C_T _value. The differences in the C_T _values of *NlFoxA *or *NlVg *and the corresponding internal control β-actin (ΔC_T_) were calculated to normalize the difference in the amount of total RNA added to the cDNA reaction mixture and the efficiency of the reverse transcription reaction. The ΔC_T _for the control sample was subtracted from the ΔC_T _of the challenged sample. The difference was expressed as a ΔΔC_T _value that allowed comparing the expressions of target genes in the challenged sample relative to the control. The expression level of *NlFoxA *or *NlVg *were calculated by 2^-ΔΔCT^[52], and the value represented an n-fold difference against the control. All the assays was guided rigidly with MIQE Precis [53].

To investigate the expression pattern of different developmental phases and tissues, we isolated total RNA from the 1^st ^to 5^th ^instar nymphs and from 1- to 10-day-old brachypterous female adults. The ovary, fat body, midgut, Malpighian tubules, cuticle, and muscle of 3^rd ^brachypterous female adults were carefully collected as described above. The specimen and tissues were rinsed in 1× PBS buffer several times, and each tissue was pooled from 15-80 individuals. All samples were used for reverse transcription to obtain the first-strand cDNA as above. The primer pairs Q *Nl*FoxAF/Q *Nl*FoxAR or Q *Nl*VgF/Q *Nl*VgR (Table 1) were designed to determine the relative expression of *NlFoxA *or *NlVg*. Each reaction mixture was done in a final volume of 10 μl containing 1 μl of the cDNA sample (or standard), 0.2 μl (10 pmoles/μl) of each primer and 5 μl of SYBR premix Ex Taq. After 10 s of an initial denaturation at 95°C, the cycling protocol consisted of 40 cycles of denaturation at 95°C for 10 s, annealing at 58°C for 20 s, and elongation at 72°C for 25 s. A *β-ACTIN *(EU179846) cDNA fragment was amplified with ACTIN-F and ACTIN-R primers (Table 1) as an internal control. The quantity of transcripts was estimated from a standard line derived from 10-fold serial dilutions of cDNA pooled from ten individuals of 2^nd ^brachypterous female adults.

For RT-PCR analysis, amplification was performed using specific primers Q *Nl*FoxAF/Q *Nl*FoxAFR and ACTIN-F/ACTIN-R (Table 1) by denaturing at 95°C for 5 min, followed by 28 cycles of 95°C for 30 s, 58°C for 30 s and 72°C for 40 s, with a final extension at 72°C for 10 min. Each PCR product (6 μl) was electrophoresed and detected by ethidium bromide staining.

### *Nl*Vg polyclonal antibody generation

A cDNA fragment containing *Nl*Vg partial sequence (4520-5306 bp) was amplified with two primers *Nl*Vg-EF and *Nl*Vg-ER (Table 1), which contain the restriction sites *Xho*I and *Nco*I, respectively. The PCR product was excised with *Xho*I and *Nco*I, and then subcloned into the pET32a vector (pET-*Nl*Vg). The recombinant *Nl*Vg protein was expressed in BL21 cells induced by IPTG. The *E. coli *pellet was solubilized in 6 M urea in 50 mM Tri-Cl buffer, pH 8.0, then purified with a Ni-NTA column (GE Healthcare). Purified recombinant *Nl*Vg protein was used to immunize rabbits as described previously [54]. The sera of the immunized rabbits were collected as anti-*Nl*Vg sera.

### RNA interference

To generate double-stranded RNA (dsRNA), a 432-bp (831-1263 bp) (*NlFoxA*) and a 600-bp (5252-5852 bp) (*NlVg) *fragment templates were amplified, as previously described, by PCR using *NlFoxA *and *Nl*Vg cDNAs as templates, with forward and reverse primers containing the T7 primer sequence at the 5' ends (Table 1). The amplification reactions protocol comprised 38 cycles of 95°C for 35 s, 55°C for 40 s and 72°C for 60 s, with a final extension step of 72°C for 10 min. The sequence was verified by sequencing (Invitrogen company, Shanghai, China). The *GFP *gene (ACY56286) was used as a control dsRNA. The PCR primers GFP-F and GFP-R were used to amplify the *GFP *fragment (688 bp) (Table 1), and dsRNA corresponding to *NlFoxA *gene (ds *Nl*FoxA) and *Nl*Vg gene (ds *Nl*Vg) were prepared according to the methods established in our laboratory [55]. The T7 RiboMAX™ Express RNAi System (Promega, USA) was used for the synthesis. To deliver dsRNA into the body of BPHs, BPHs were reared on an artificial diet [56], with some modifications to the rearing protocol. We used glass cylinders measuring 9.0 cm in length and 2.0 cm in diameter as feeding chambers. The dsRNA solution was added to the artificial diet, held between two layers of stretched Parafilm M that were enclosed at the two open ends of the chamber. The diet (each end was loaded 10 μL)was renewed every day. The dsRNA concentrations were designated as high dose (0.5 μg/μl) and low dose (0.1 μg/μl), as previously established [36]. The cylinders were covered with a piece of black cotton cloth, but the two ends where the artificial diet was placed were exposed to light. Insects could feed on the diets by puncturing the inner Parafilm M membrane of the diet pouch. All insects were transferred into chambers and pre-reared on artificial diets for 1 day before initiation of the assays. Then fifteen 3^rd ^instar individuals were transferred into each chamber, and every three chambers were used in each set of triplicate repetitions. The rearing experiments were carried out in a growth cabinet with a humidifier at 27°C, using 90% RH and a 16:8-h light:dark photoperiod.

### Bioassay and data analysis

To analyze the influence of *NlFoxA *and *NlVg *genes on fecundity of N. *lugens*, we planted rice plants in small basins 20 cm deep by 25 cm in diameter, each covered with two ends of an opened transparent plastic cylinder. The cylinder was 70 cm in height and 20 cm in diameter, with two small windows in the wall, leaving a 12-cm gap from the bottom. The top and windows were enclosed with a piece of nylon mesh to prevent the insects from moving in or out. Ten days later, all nymphs were transferred to rice plants that are tillering, the newly emerged brachypterous adults were collected, and each female was matched with one male from each basin of the rice plants. Spawning lasted 15 days, and the parents were removed after oviposition. The offspring were counted by CO_2 _anesthetization 8 days after the parents were removed.

The efficiency of *NlFoxA *gene silencing on the effect of knockdown on *NlVg *and fecundity in BPH were assessed. Experiments employed different concentrations and the same feeding protocol as described above. The brachypterous adults were collected from 1 to 8 days following emergence. Three synchronous larvae were sampled randomly every day in replicates of three. The relative mRNA expression level was analysed by RT-qPCR. Primers and reaction conditions were the same as described above.

### Ovary dissection and microscopy

To determine the impact of gene silencing on the development of ovarioles in BPH, the feeding and raising protocols were the same as described above. The ovaries were dissected from the 3^rd ^day of brachypterous female adult *N. lugens *in 1× phosphate buffered saline (PBS) (137 mM NaCl; 2.68 mM KCl; 1.47 mM KH_2_PO_4_; and 8.10 mM Na_2_HPO4, pH 7.0), followed by fixation in 3.8% formaldehyde in 1× PBS for 20 minutes at room temperature. Dissected ovaries were washed three times for 10 minutes with 0.2% Triton-X 100 (Sigma, USA) in 1× PBS. After washing, ovaries were photographed with a Leica DMR connected to a Fuji FinePix S2 Pro digital camera(Germanny).

### Enzyme-linked immunosorbent assay (ELISA) and western blot analysis

Variations of Vg protein caused by RNAi were determined through ELISA and western blot analysis. Total proteins were extracted from fifteen individuals' ovaries of BPH in four different treatment groups. The same total amount of protein was coated on microtiter plates. Briefly, these total proteins were diluted to 0.5 mg/mL with carbonate buffered solution (0.05 M, pH 9.6) and used to coat 96-well microplates at 100 μL/well. Following overnight incubation at 4°C, the plates were washed three times with PBS-T (PBS containing 0.05% Tween 20, USA) and blocked with 5% skim milk in PBS (100 μL/well). After incubation at 37°C for 1 h, the plates were washed 3 times with PBST. The rabbit polyclonal antibodies were serially diluted through the wells using PBS, and the plates were incubated for 1 h at 37°C. After a subsequent wash step, an enzyme-labeled second antibody (goat-anti-rabbit labeled with horse radish peroxidase, GAM-HRP (BOSTER, Wuhan, China) was diluted 1:8000 with PBST and added to the plates (100 μL/well). The plates were incubated for 1 h at 37°C and washed 4 times with PBST (200 μL/well). The substrate solution (TMB buffer, pH 5.5) was then added at 100 μL/well to each well. After 10 min at room temperature, the reaction was terminated by 50 μL/well 2 M H_2_SO_4_. The absorbance was measured at 450 nm. PBST (0.1 M, pH 7.4) instead of polyclonal antibodies was used in the blank control. The value of OD_450 _indicates the concentration of *Nl*Vg protein.

Western-blotting analysis were modified according to the methods from the previously described [57]. Briefly, totally 300 μg ovaries proteins (as above) were separated on a 12% SDS-PAGE gel, transferred to NT membranes (PVDF, Bio-Rad), immunoblotted with anti-Vg serum (diluted 1:5000) and an IgG goat anti-rabbit antibody conjugated with HRP was used for secondary antibody (BOSTER, Wuhan, China, 1:5000 dilution), finally visualized by ECL (enhanced chemiluminescence).

### Statistical analysis

The results are expressed as the means (± S.E.M.). SPSS 13.0 software was used to perform *t*-tests to identify significant differences at a 95% confidence level (p < 0.05).

## Authors' contributions

XD has completed most of the experiment and proofread, developed the concept and wrote the manuscript. YZ has reared the PBH and complete partial experiment. JZ, ZS, JC and JC were involved in the data and sequence analysis. WZ has designed the experiment and polished the manuscript. All the authors have approved the final form of the manuscript.
